# Supplementary figures and images for: Interactions of Severe Acute Respiratory Syndrome Coronavirus 2 Protein E With Cell Junctions and Polarity PSD-95/Dlg/ZO-1-Containing Proteins
Source: Front Microbiol. 2022 Feb 23;13:829094. doi: 10.3389/fmicb.2022.829094 (PMC8909127; doi:10.3389/fmicb.2022.829094)

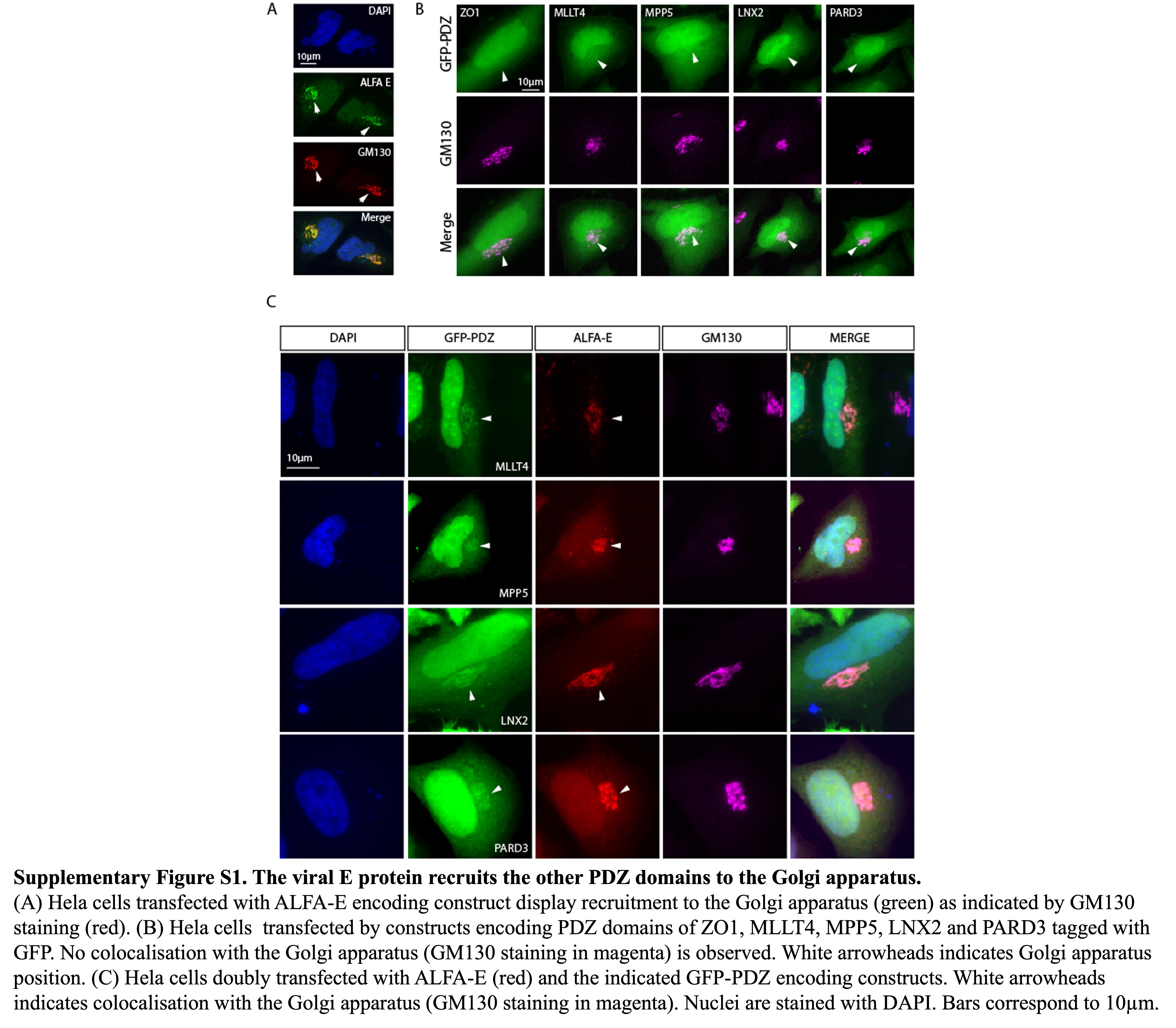

Supplement: Supplementary file 1 [file Image_1.JPEG]

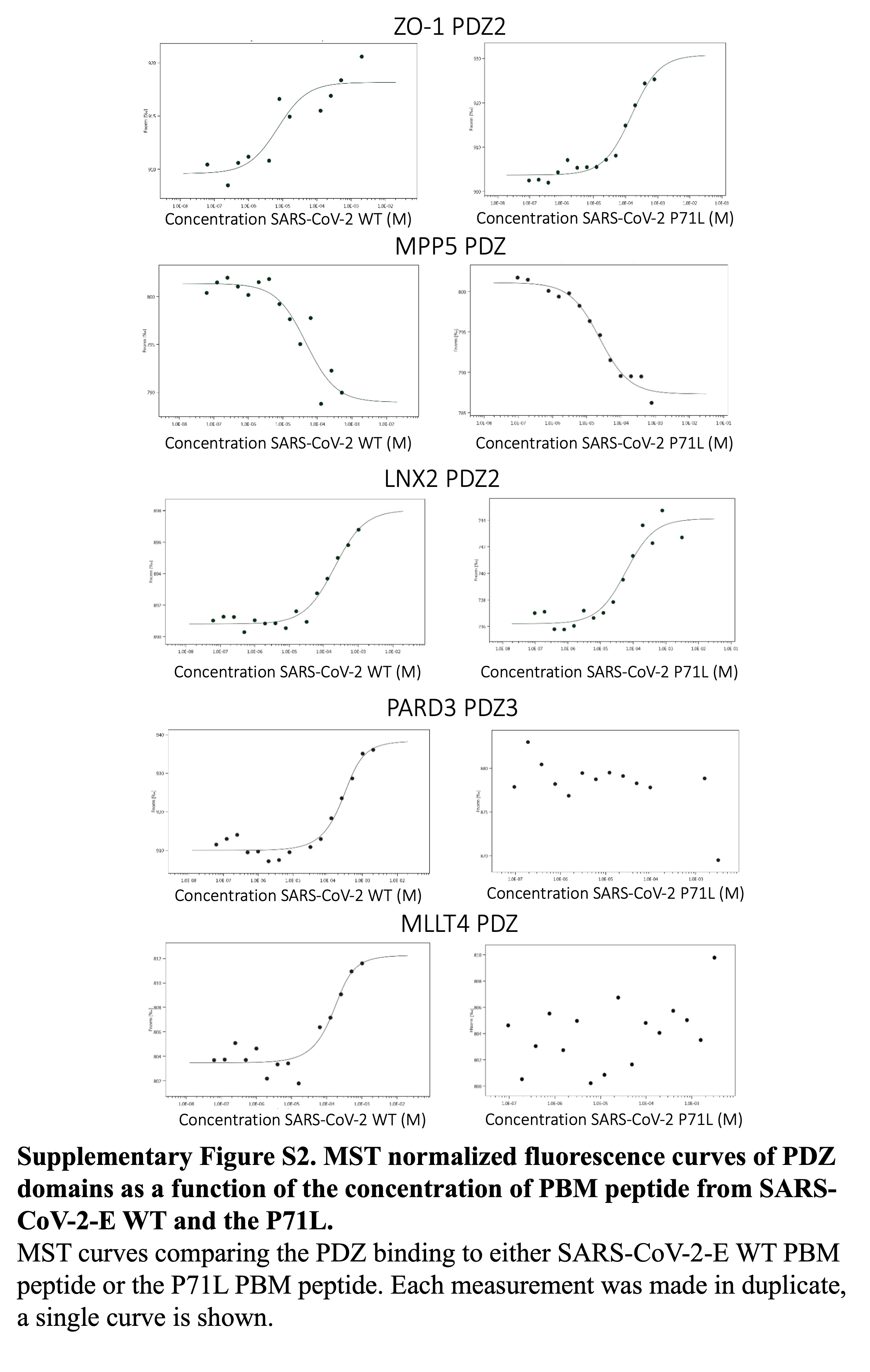

Supplement: Supplementary file 2 [file Image_2.JPEG]

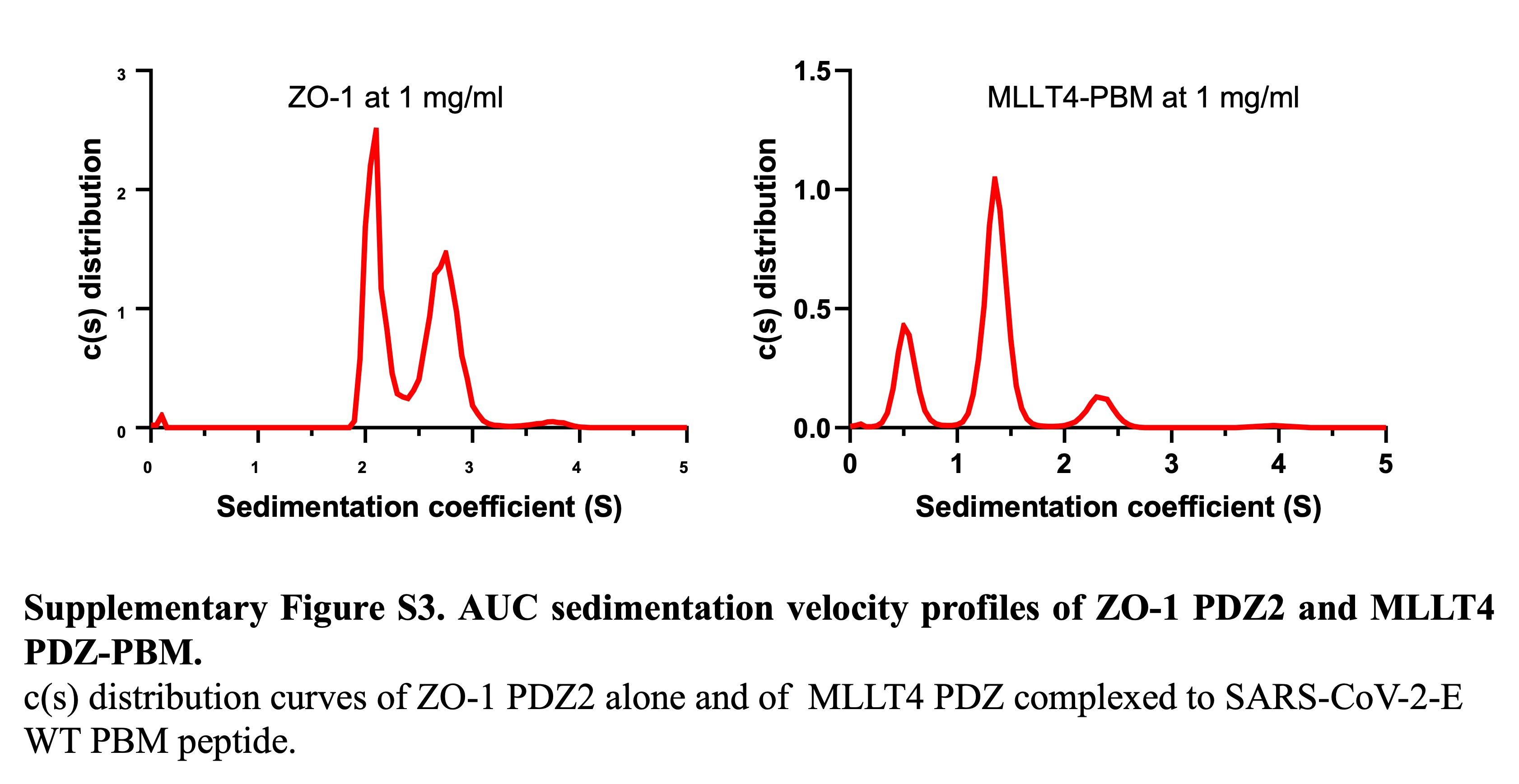

Supplement: Supplementary file 3 [file Image_3.JPEG]
